# Supplementary material for: Invasive crayfish does not influence spawning microhabitat selection of brown frogs
Source: PeerJ. 2020 Apr 15;8:e8985. doi: 10.7717/peerj.8985 (PMC7166042; doi:10.7717/peerj.8985)
Supplement: Supplemental Information 3 — Tests of heterogeneity of variance of microhabitat features between invaded and uninvaded sites. Significant likelihood ratio tests indicate that variance was significantly heterogeneous among groups, therefore mixed models with heterogeneous variance were used for analyses. [file peerj-08-8985-s003.docx]

| Species | Independent variable | Variance | | χ^2^ | *P* |
| --- | --- | --- | --- | --- | --- |
|  |  | Without crayfish | With crayfish |  |  |
| *R.latastei* | Distance from nearest clutch | 81.9 | 1307.2 | 4.16 | **0.04** |
|  | Water depth at the spawning site | 218.5 | 84.7 | 3.87 | **0.049** |
|  | Distance from edge | 15733 | 16566 | 0.16 | 0.68 |
| *R.dalmatina* | Distance from nearest clutch | 3878 | 2395 | 89.86 | **<0.0001** |
|  | Water depth at the spawning site | 60.7 | 88.6 | 3.15 | 0.07 |
|  | Distance from edge | 29304 | 35898 | 23.17 | **<0.0001** |
